# Editorial Department

**Published:** 1856-02

**Authors:** 


The "Buffalo Daily Express" has recently taken the charities of the medical profession under its supervision. It relates a lamentable case, wherein a physician who had grown wealthy, refused to go and reduce a fracture of the fore-arm for a poor woman. Shocking! particularly so, as the county authorities claim to pay to certain members of the profession a *very* liberal remuneration for taking care of such cases. Another physician was found, who went without inquiry and dressed the fracture. Our position on the matter is, that both of these gentlemen acted entirely right; the one to refuse if it was inconvenient to go, if he was not engaged in surgical practice, or if he pays his share of taxes toward the support of the poor, and the other to go if he had the time and the disposition to do a charitable act, while the county was paying another man on the supposition that he performs such services.

It is immensely easy to be charitable in an easy chair in the solitude of one'ssanctum; but for one who, like the writer of this and hundreds others of his brethren, has never, in years of practice, refused a call however humble; one who has done enough of this sort of thing to have lost all the romance with which he commenced his never-ending succession of charities; one who out of his own poverty has yearly given more in time, and medicines, and watchings by night, than would equal the collected charities of all the editors of all the daily papers in town for the last ten years; it is *not* immensely pleasant to see a professional man held up to scorn because he chooses for once to throw a charity on the shoulders where it of right belongs.

